# Nurses’ and patients’ communication in smoking cessation at nurse-led COPD clinics in primary health care

**DOI:** 10.3402/ecrj.v2.27915

**Published:** 2015-08-07

**Authors:** Eva Österlund Efraimsson, Birgitta Klang, Anna Ehrenberg, Kjell Larsson, Bjöörn Fossum, Lena Olai

**Affiliations:** 1 Division of Nursing, Department of Neurobiology, Care Sciences and Society, Karolinska Institutet, Stockholm, Sweden; 2 Centre for Clinical Research, Dalarna, Falun, Sweden; 3 Dalarna County Council, Norslund Primary Health Care Centre, Falun, Sweden; 4 School of Health and Social Sciences, Dalarna University, Falun, Sweden; 5 Red Cross University College of Nursing, Stockholm, Sweden; 6 National Institute of Environmental Medicine, Karolinska Institutet, Stockholm, Sweden; 7 Department of Clinical Science and Education, Södersjukhuset, Karolinska Institutet, Stockholm, Sweden; 8 Sophiahemmet University, Stockholm, Sweden; 9 Department of Public Health and Caring Sciences, Family Medicine and Preventive Medicine Section, Uppsala University, Uppsala, Sweden

**Keywords:** change talk, chronic obstructive pulmonary disease, motivational interviewing, nurse-led clinics, smoking cessation, videotaped consultation

## Abstract

**Background:**

Smokers with chronic obstructive pulmonary disease (COPD) have high nicotine dependence making it difficult to quit smoking. Motivational interviewing (MI) is a method that is used in stimulating motivation and behavioral changes.

**Objective:**

To describe smoking cessation communication between patients and registered nurses trained in MI in COPD nurse-led clinics in Swedish primary health care.

**Methods:**

A prospective observational study with structured quantitative content analyses of the communication between six nurses with basic education in MI and 13 patients in non-smoking consultations.

**Results:**

Only to a small extent did nurses’ evoke patients’ reasons for change, stimulate collaboration, and support patients’ autonomy. Nurses provided information, asked closed questions, and made simple reflections. Patients’ communication was mainly neutral and focusing on reasons for and against smoking. It was uncommon for patients to be committed and take steps toward smoking cessation.

**Conclusion:**

The nurses did not adhere to the principles of MI in smoking cessation, and the patients focused to a limited extent on how to quit smoking.

**Practice implications:**

To make patients more active, the nurses need more education and continuous training in motivational communication.

Chronic obstructive pulmonary disease (COPD) is a preventable and treatable disease, caused by smoking or by occupational exposure, characterized by accelerated decline in lung function with symptoms such as coughing, phlegm, dyspnoea, and fatigue (1). COPD, which is one of the leading causes of morbidity and mortality worldwide, has during the past decade become more recognized among the general population (2). This has involved increased awareness of the importance of quitting smoking and of the possibility of seeking care for smoking cessation. However, the combination of age, many years of smoking, and severe nicotine addiction makes it difficult for patients with COPD to quit smoking (3–6), increasing their ambivalence toward smoking cessation and their need for qualified support (7, 8).

Motivational interviewing (MI) is a clinical communication method used for qualified support to resolve ambivalence about change by exploring and resolving motivation to increase patients’ engagement in treatment. MI is described as collaborative, evocative, and supportive of patients’ autonomy to reinforce patients’ motivation for change. MI is based on four guiding principles: to resist the righting reflex, to understand and explore the patient's own motivation to listen with empathy, to empower the patients, and encourage hope and optimism. Patients should be viewed as experts on their own ability to minimize resistance to change, and thereby enhance their motivation (9). Motivated patients are anticipated to participate more actively in behavior change, engage more in self-disclosure, and assume greater responsibility in their efforts toward change (10). Motivation involves recognizing a problem, searching for a way to change, and implementing and maintaining that change.

In MI, the professional's goal is to help the patients to face feelings of ambivalence, to evaluate and resolve them, and to find the motivation to move forward toward the ultimate target behavior (11).

Internationally, MI has been used progressively in medicine and public health mostly in different groups of individuals with unhealthy life styles. A number of systematic reviews and meta-analysis have tried to establish evidence for the effects of MI. Weak evidence has been found for effects on patients’ self-monitoring, confidence in change, approach to treatment, health behavior change, engagement, and reduced risk behaviors (12–20). A recent review showed a modest but significant increase in smoking cessation in a general population of patients when primary care physicians used MI, compared to usual care. Shorter sessions showed better effect than longer, and MI provided by registered nurses (RNs) was less effective (21). In Swedish COPD clinics, RNs provide self-management education and support patients in quitting smoking. In spite of the lack of strong supporting evidence, MI is often used for this purpose and at least 60% of all RNs in Swedish primary health care (PHC) have undertaken basic education in MI (2 days and 1 day for follow-up) (22).

In systematic reviews of MI, problems with weak designs are reported and more studies are called for. Furthermore, MI appears to be difficult to implement with high fidelity and requires practice, feedback, and coaching over time (10, 12–17). For a more complete picture of smoking cessation communication, there is also a need to study patients’ communication, interaction between patients and RNs, and the development of communication over time. Therefore, the aim of this study was to describe smoking cessation communication between patients and RNs trained in MI in COPD nurse-led clinics in Swedish PHC.

## Methods

### Design

A prospective observational study with structured quantitative content analyses of the communication between RNs and patients in smoking cessation based on the Motivational Interviewing Treatment Integrity (MITI) and the Client Language Assessment in Motivational Interviewing (CLAMI) scales.

### Setting and sample

This study context was nurse-led COPD clinics in six PHC clinics located in rural and urban areas in the central and southern parts of Sweden.

The inclusion criteria for the clinics (in compliance with the national criteria for asthma and COPD clinics in Swedish PHC, 1998) were: specially trained RNs in asthma and COPD, who spend more than 0.5 h/week/1,000 inhabitants caring for patients with asthma and COPD, and a physician responsible for the unit. The RNs were required to have at least 2 years’ experience as COPD nurses and basic training in MI (Table 1). A convenience sample of RNs, who consented to have their patient consultations observed, contributed with one to four patients consecutively selected upon referral. One-hour pre-scheduled appointments, spirometry before and after bronchodilatation, pulsoximetry, and structured assessment with patient education physiology and pathophysiology, treatments, and self-care strategies (1, 18, 23) were performed. Patients were included if they were smokers, had respiratory symptoms, and were referred to the COPD clinic for assessment (Table 2).

**Table 1:** Characteristics of nurses (*n*=6)

Age, years	
Mean (range)	51 (45–60)
Gender	
Female	6
Numbers of nurses with university specialist education in public health nursing	
0 ECTS credits	2
7.5 ECTS credits	4
Numbers of nurses with university education in COPD	
15 ECTS credits	5
22.5 ECTS credits	1
Years working as asthma/COPD nurse	
Mean (SD, range)	10 (3.5, 5–14)
Days of MI-based education in smoking cessation	
Mean (SD, range)	4 (2, 2–7)

ECTS=European Credit Transfer System; COPD=chronic obstructive pulmonary disease; SD=standard deviation; MI=motivational interviewing.

**Table 2:** Characteristics of the patients (*n*=13)

	Frequency	Female/male patients
Patients	13	11/2
Age, years		
Mean (SD)	52(14)	49/74
Marital status		
Living together	7	5/2
Occupation		
Employed	9	9/0
Retired	4	2/2
Education		
Compulsory school	4	3/1
Upper secondary school	9	8/1
University level	0	0/0
Severity of COPD (GOLD criteria)		
No COPD	2	2/0
Stage 1	7	7/0
Stage 2	2	1/1
Stage 3	2	1/1
Stage 4	0	0/0

SD=standard deviation; COPD=chronic obstructive pulmonary disease; GOLD=global initiative for chronic obstructive lung disease.

### Procedure

A questionnaire covering demographic data and smoking habits was filled in by the patients, before the first and after the third consultation, 3–8 months apart. A video camera was running during the whole consultation, but the investigator was not present during the consultation.

### Instruments for data analysis

The behavioral coding system, MITI scale (19), and the CLAMI segment (20) were used in the analysis of the videotapes. MITI assesses the practitioner's use of MI, with the RNs utterances being the unit of analysis, and CLAMI assesses the patient's talk within a MI session, with the patient utterances being the unit of analysis. The coders assess the RNs’ and patients’ verbal communication with emphasis on a specific coding task, the Target Behavior Change (TBC), namely the verbal smoking cessation communication, as exemplified in Appendix A1.

#### Motivational Interviewing Treatment Integrity

MITI has proved to be a reliable tool for evaluating the use and training of MI (24–27) and has shown good validity with regard to communication behavior and MI skill development over time (15, 28). Two coding procedures are applied in MITI, ‘global scores’ and ‘behavioral codes’. The MITI global scores describe how the RN shows *empathy*, evokes patient's reasons for change, fosters *collaboration*, and supports patient's *autonomy*, and finally how the RN maintains appropriate focus on the smoking cessation communication (*direction*) on a 5-point Likert-scale, ranging from 1 (low) to 5 (high). All dimensions were assessed as individual parameters, while Evocation, Collaboration, and Autonomy Support were also averaged together, yielding a ‘MI-spirit’ score indicating the general impressions of the three parameters (Appendix A2) (19). Behavior codes: Questions, Reflections, Giving information, MI Adherent, and MI Non-adherent, imply registrations of the frequency of specific utterances during the recorded session. The Questions code includes closed and open questions, and the Reflection code includes simple and complex reflections (Appendix A3). The coder does not judge the quality or appropriateness of the utterances, but simply counts the number of different utterances exhibited by the RN.

#### Client Language Assessment in Motivational Interviewing

Reliability data for CLAMI showed good to excellent interrater reliability for all CLAMI variables (29). Within CLAMI, language moving in the direction of change is termed ‘change talk’(+), while language indicating a movement away from change, is called ‘sustain talk’(−). Both change- and sustain talk were coded in four categories: 1) reason (sub-codes: desire, ability, and need), 2) other, 3) taking steps, and 4) commitment. Every time one of the categories occurs in patient talk, the category is recorded as change (+) or sustain (−) talk. If a patient's talk about smoking is neither toward nor away from the TBC, it is coded as a fifth category, Follow/Neutral (Appendix A4) (19).

### Coding

The coding was undertaken at the Motivational Interviewing Coding (MIC) Laboratory at Karolinska Institutet in Stockholm by three qualified coders. One coder listened to the entire consultation for both MITI global score and behavior codes (19) and another for the CLAMI categories. The coders had more than 80 h of initial training, divided equally between MITI and CLAMI, in accordance with the current recommendations followed by 3-h training sessions every fortnight to achieve adequate interrater reliability, precision, and quality in the coding.

### Reliability

To safeguard reliability, five video-recorded consultations were independently coded by two coders for both MITI and CLAMI, and interrater reliability was calculated with the intraclass coefficient (ICC). ICC takes into account the frequency of equal variable ratings for the coders, as well as possible systematic differences between the coders. For MITI (global scores and behavior codes) agreement was excellent (0.9–1.0), for CLAMI ‘taking steps’ acceptable agreement (0.5), and for the remaining CLAMI categories ICC ranged from 0.8 to 1.0, indicating excellent agreement (30).

### Statistical analysis

Statistical analyses, including descriptive statistics such as summations, percentages, mean (m), ranges, and standard deviations (SD), were performed using Statistical Package for Social Sciences (SPSS) 17.0. Coded data for the MITI global scores were treated as ordinal data. The MITI behavior codes and CLAMI categories data were treated as interval data.

### Ethics

Local managers, RNs, and patients were provided with oral and written information about the study and informed consent was received. To guarantee confidentiality, only the researchers and coders had access to the videotaped consultations (31). The study was approved by the Research Ethics Committee at Karolinska Institutet, Stockholm, Sweden.

## Results

The study included 26 consultations with 6 female RNs and 13 patients who were smokers in session one and three out of three visits, March 2006 to April 2007 at nurse-led COPD clinics. The mean duration of the first consultations was 43 min (SD 9.0), of which 15 min (35%; SD 10.4) were used for smoking cessation communication. The third consultation had a mean duration of 33 min (SD 6.7), of which 11 min (33%; SD 6.3) were focused on smoking cessation.

### Nurses talk about smoking cessation

The global score Direction, indicating RNs’ focus on smoking cessation, rated the highest (5) in all 26 consultations, while the remaining global scores rated between 1 (low) and 3 (medium) (Table 3).

**Table 3:** The MITI scale: judgment of global scores for each consultation (1=low – 5=high)

	Global scores					
	
	Evocation	Collaboration	Autonomy support	Direction	Empathy	MI-spirit
	
Consultation	First consultation (*n*=13)/third consultation (*n*=13)					
A	1/1	1/1	2/2	5/5	1/2	1.3/1.3
B	1/2	2/3	2/3	5/5	3/3	1.7/2.7
C	2/2	3/2	3/2	5/5	3/3	2.7/2.0
D	1/1	2/3	2/3	5/5	2/3	1.7/2.3
E	2/1	2/2	2/1	5/5	2/2	2.0/1.3
F	1/1	1/2	1/2	5/5	1/2	1.0/1.7
G	2/1	1/2	2/3	5/5	2/2	1.7/2.0
H	3/1	2/2	2/2	5/5	2/2	2.3/1.7
I	1/1	2/2	2/2	5/5	2/2	1.7/1.7
J	1/2	1/2	2/2	5/5	2/2	1.3/2.0
K	1/2	2/2	2/2	5/5	2/2	1.7/2.0
L	1/1	2/2	2/2	5/5	2/2	1.7/1.7
M	1/1	2/1	2/2	5/5	2/2	1.7/1.3
Total	18/17	23/26	26/28	65/65	26/29	22.4/23.7
Mean (m)	1.4/1.3	1.8/2.0	2.0/2.2	5.0/5.0	2.0/2.2	1.6/1.8
Standard deviation (SD)	0.67/0.49	0.62/0.51	0.43/0.58	0/0	0.58/0.44	0.43/0.40

MITI=Motivational Interviewing Treatment Integrity; MI=motivational interviewing; SD=standard deviation.

**Fig. 1:**
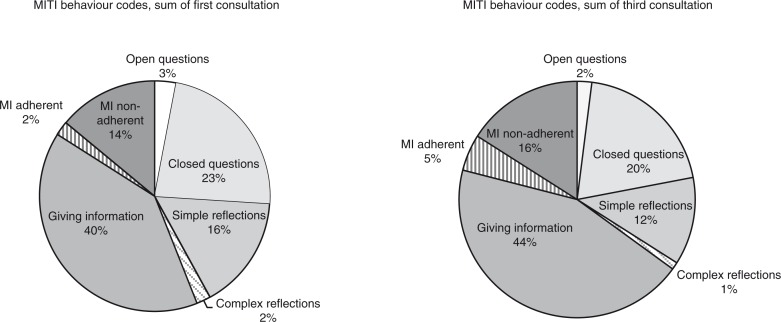
MITI behavior codes, sum of first respectively third consultation.

The result of the MITI behavior codes showed that it was common for the RNs to ask *closed questions*, yes/no, mean 8.2 and 6.2, respectively, and to reflect or summarize patients’ statements without adding additional meaning to what the patients had said (*simple reflections*), mean 5.6 and 3.9, respectively. Most common was to provide information, educate, and give feedback (*giving information*) about smoking cessation during the consultation, mean 14.2 and 14.8, respectively. Further, the RNs gave advice without permission, confronted patients, gave orders, commanded, or made imperatives (*MI-non-adherent behavior*), mean 4.9 and 5.0, respectively. It was uncommon for RNs to ask *open questions* that allowed a wide range of answers, mean 1.2 and 0.8, respectively. The RNs seldom reflected on or summarized what the patients had said with a substantial or deeper meaning (*complex reflections*), mean 0.9 and 0.4, respectively, or used *MI-adherent behavior*, with a mean of 0.9 and 1.5, respectively (Table 4). The distribution of the MITI behavior codes showed that the RNs most often were giving information to the patients, followed by closed questions (Fig. 1).

**Table 4:** The MITI scale: frequencies of behavior codes for each consultation

	Behavior codes						
	
	Giving information	MI adherent	MI Non-adherent	Open question	Closed question	Simple reflections	Complex reflections
	
Consultation	First consultation (*n*=13)/Third consultation (*n*=13)						
A	33/23	1/2	9/8	2/2	20/3	10/4	1/0
B	6/4	0/1	1/2	0/0	4/2	6/0	0/0
C	17/6	1/4	0/8	0/2	4/6	2/6	3/2
D	6/6	1/1	3/0	0/0	4/2	2/1	0/0
E	18/19	1/1	7/10	2/1	11/7	7/3	2/0
F	10/10	1/0	5/2	1/3	7/7	7/2	0/2
G	14/17	1/6	5/5	4/1	11/11	4/4	0/1
H	22/25	0/2	12/10	4/0	8/8	8/6	1/0
I	5/12	0/1	4/7	0/0	8/13	4/13	0/0
J	15/8	1/0	6/3	0/0	7/4	9/2	1/0
K	13/12	0/0	3/3	1/0	9/3	5/2	1/0
L	5/1	0/1	3/2	0/0	8/0	4/0	1/0
M	20/34	4/0	6/5	2/1	5/14	5/7	1/0
Total	184/177	11/19	64/65	16/8	106/80	73/50	11/5
Mean (m)	14.2/14.8	0.9/1.5	4.9/5.0	1.2/0.8	8.2/6.2	5.6/3.9	0.9/0.4
Standard deviation (SD)	8.13/9.44	1.07/1.76	3.2/3.32	1.48/1.01	4.30/4.41	2.50/3.55	0.90/0.77

MITI=Motivational Interviewing Treatment Integrity; MI=motivational interviewing.

### Patients’ talk about their smoking

**Fig. 2:**
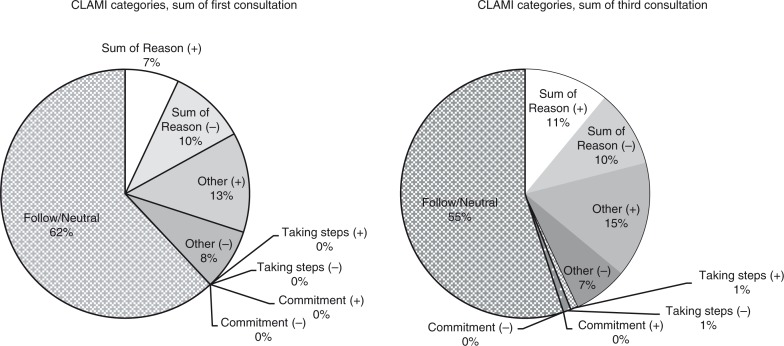
CLAMI categories, sum of first respectively third consultation.

The results from the CLAMI categories showed that on average 60% of patients’ utterances followed the RNs’ talk with replies such as ‘Sure’/‘OK’ and were coded in the category *Follow/Neutral*, with a mean frequency of 23.5 in the first and 20.2 in the third consultation. About 40% of patients’ utterances were divided between the remaining CLAMI categories. Sum of reason (reason, desire, ability, and need) covers patients’ utterances about rationale and motivation and was exhibited in change talk with a mean of 2.7 in the first, 3.9 in the third consultation, and in sustain talk a mean of 3.6 and 3.7, respectively. The category *Other* (problem identification, minimization of problem, and hypothetical language) showed in change talk a mean of 5.0 and 5.3, respectively and in sustain talk 3.2 and 2.4, respectively. Concrete steps toward smoking cessation expressed by the patient, *Taking Steps*, showed in change talk a mean of 0.1 and 0.5, respectively. Corresponding figures for sustain talk were 0.1 and 0.2, respectively. In *Commitment* language, agreement, intention, or obligation regarding smoking cessation, no utterances were made in the first consultation. In the third consultation change talk showed a mean of 0.2 and sustain talk a mean of 0.1 (Table 5, Fig. 2).

**Table 5:** Frequencies of CLAMI categories for each consultation

	Change talk (+)									Sustain talk (−)									
		
	Reason	Desire	Ability	Need	Sum reason^a^	Other	Taking steps	Commitment	Total	Reason	Desire	Ability	Need	Sum reason^b^	Other	Taking steps	Commitment	Total	Follow-neutral
	
Consultation	First consultation (*n*=13)/Third consultation (*n*=13)																		
A	2/0	0/0	2/1	0/0	4/1	5/1	0/0	0/0	9/2	1/2	0/0	0/0	0/0	1/2	5/6	0/0	0/1	6/9	44/20
B	0/0	0/0	0/0	0/0	0/0	1/0	1/1	0/0	2/1	2/0	0/0	0/0	0/0	2/0	1/0	0/0	0/0	3/0	9/7
C	1/1	0/0	0/0	0/0	1/1	4/5	0/0	0/0	5/6	3/3	0/0	0/0	1/0	4/4	4/3	0/0	0/0	8/7	18/29
D	0/0	0/0	0/0	0/0	0/0	1/0	0/0	0/0	1/0	3/3	0/0	0/2	0/1	3/5	2/1	1/0	0/0	6/6	12/8
E	6/3	0/0	1/1	0/4	7/8	6/8	0/0	0/0	13/16	12/2	0/0	0/5	0/0	12/7	6/4	0/0	0/0	18/11	31/41
F	3/4	0/0	2/0	1/1	6/5	9/0	0/1	0/0	15/6	4/3	0/0	0/0	0/0	4/4	0/1	0/1	0/0	4/6	25/12
G	1/4	0/0	0/0	1/1	2/9	7/9	0/1	0/1	9/21	0/0	0/0	0/0	0/1	0/0	1/0	0/1	0/0	1/1	31/14
H	1/10	0/0	2/1	0/0	3/12	11/13	0/2	0/0	14/27	4/11	0/0	0/1	1/0	5/12	7/5	0/0	0/0	12/17	37/43
I	0/3	0/1	0/0	0/0	0/4	0/6	0/0	0/0	0/10	0/1	0/0	0/0	0/0	0/1	0/5	0/0	0/0	0/6	14/21
J	3/3	2/0	0/0	0/0	5/3	4/2	0/1	0/0	9/6	8/2	0/0	0/0	0/0	8/2	6/1	0/0	0/0	14/3	22./20
K	0/2	0/1	0/0	0/0	0/3	1/3	0/0	0/0	1/6	1/2	0/0	0/0	0/0	1/2	5/0	0/1	0/0	6/3	18/14
L	1/2	0/0	1/0	0/0	2/2	6/6	0/1	0/0	8/9	0/2	0/0	0/1	1/0	1/3	2/0	0/0	0/0	3/3	9/4
M	4/2	1/1	0/0	0/0	5/3	10/16	0/0	0/1	15/20	5/6	0/0	1/0	0/0	6/6	2/5	0/0	0/0	8/11	36/30
Total	22/34	3/3	8/3	2/6	35/51	65/69	1/7	0/2	101/130	43/37	0/0	1/9	3/2	47/48	41/31	1/3	0/1	89/83	306/263
Mean(m)	1.7/2.6	0.2/0.2	0.6/0.2	2/0.8	2.7/3.9	5/5.3	0.1/0.5	0/0.2	7.8/9.8	3.3/2.9	0/0	0.1/0.7	0.2/0.2	3.6/3.7	3.2/2.4	0.1/0.2	0/0.1	6.7/9.8	23.5/20.2
Standard deviation(SD)	1.84/2.63	0.60/0.44	0.87/0.44	0.38/1.48	2.5/3.68	3.63/5.1	0.28/0.66	0/0.38	5.54/8.2	3.5/2.88	0/0	0.28/1.44	0.44/0.38	3.50/3.3	2.44/2.33	0.28/0.44	0/0.28	5.21/4.68	11.47/12.44

a Sum reason positive (+)=reason (+), desire (+), ability (+) need (+)

b sum reason negative (−)=reason (−), desire (−), ability (−) need (−).

CLAMI=Client Language Assessment in Motivational Interviewing.

The smoking cessation communication between patients and RNs did not seem to develop over time. Patients in the third consultation did not express more reason for change or commitment toward smoking cessation, as compared to the first consultation.

## Discussion

In this prospective observational study, the communication between RNs and patients in smoking cessation was analyzed using the MITI and the CLAMI scales.

Although MI has not shown strong evidence for smoking cessation provided by RNs (21), this communication strategy has been extensively used in public health over the last 20 years (32), and it has been promoted to be used by RNs in Swedish PHC (22). The RNs in the present study did not provide communication in adherence with the principles of MI, and the patients’ communication did not reflect a readiness for change, proposed as an important factor in predicting positive client outcomes, for example, smoking cessation. The patients’ talk was mostly coded as Follow/Neutral indicating that the patients responded to the RNs with nods or words of approval, as seen in other studies evaluating MI communication with CLAMI (33–35).

The findings are similar to previous findings on communication patterns between RNs and patients with various chronic conditions. In diabetes care, studies of video-recorded consultations between RNs and patients with Type 2 diabetes have been performed. One study of interaction with newly diagnosed patients showed that the communication was driven by the RNs agenda and largely ruled by extensive checklists and to a lesser extent by the needs of the patients (36). Another study showed that the RNs largely focused on providing health information based on medical jargon and to a limited extent checked for patients’ understanding (37). Studies of RNs and patients with stroke in hospital wards showed that RNs controlled the topic and flow of the communication, and posed closed questions, which resulted in an asymmetric interaction (38).

Cognitive dissonance such as reducing the conflicts between wanting to smoke and knowing that it is unhealthy by denying and avoiding information (39–41) could explain why the patients followed or were neutral toward the RNs’ claims, and why the CLAMI category Reason seldom occurred in the patients’ communication. Therefore, it is essential that the health care staff understand and address both the physical and psychological aspects of the addiction and disease, when supporting patients quitting smoking. However, the psychological burden of patients with COPD, leading to a low quality of life (42), could cause RNs to refrain from exposing the patients to possible feelings of guilt and shame. This could also explain why only a mean of 15 min in the first and 11 min in the third consultation were used for smoking cessation.

It is hypothesized that behavior change could emerge gradually over time as the relationship develops during the RN's successive guidance and encouragement of the patient (9, 11). Therefore, it is interesting that patients in the third consultation neither expressed more reason for change nor more steps and commitment toward smoking cessation, as compared to the first consultation. The similarities in the communication patterns might further indicate that the RNs had not planned the consultations with strategies for development of MI communication in mind or with support from a treatment-plan.

The conformity in RNs’ and patients’ communication in the first and third consultation could also be explained by experienced RNs employing a fixed communication strategy including giving information about the disease, and self-management, not tailored for each patient's needs, which coheres with findings from similar studies for other patient groups in other contexts (36, 38). The RNs provided a lot of information, asked mainly closed questions and exhibited a MI non-adherent behavior. This may indicate that the MI skills contrast with RNs’ traditional counseling techniques. It has been shown that the PHC nurses experience barriers to learning MI including difficulty in adjusting to a new way of communication and thinking, and changing from an authoritarian expert approach, to a person centered one (43). The barriers experienced by RNs toward learning MI are probably due to insufficient and ineffective MI training, which may be the reason for the low use of MI by the RNs in this study. They had an average of 3 days’ education in MI, although it is recognized that 3–5 days of training are insufficient and that additional continuing supervision and feedback are needed to reach MI competence (17, 44, 45).

### Strengths and limitations of the study

This study is based on a limited sample of nurse–patient consultations and, therefore, generalizability should be done with caution. However, the amount of utterances and responses from the 26 consultations was extensive, and the coding process was elaborate in order to identify all content relevant for smoking cessation communication in the video-recorded sessions.

Videotaping as a research method might influence RNs’ and patients’ communication. In this study, the participating RNs were specialized and experienced in COPD care and were aware of the purpose of the study, implying that they had an interest in doing a good job, which constituted an unavoidable selection bias. However, videotaping could be seen as a strength, facilitating the collection of data on complex interactions and behaviors in clinical consultations (46, 47). It has also been claimed that videotaping is an unobtrusive observational method, which causes limited disturbance to the consultation process, and is therefore considered to be a valid and reliable method (48).

## Conclusion and implications

This observational study indicates that scheduled consultation time with smokers at nurse-led COPD clinics in PHC was not used optimally for smoking cessation communication. In spite of the RNs’ basic training in MI, the consultations had a traditional, consultative content, with RNs providing a lot of information, asking closed questions, and patients that mostly followed or were neutral toward what the RNs had said. The communication in the first and third consultation had also similar content: there was consistency in lack of guidance that could have evoked the motivation for smoking cessation. The RNs’ talk and questions to the patients evoked only to a small extent the patients’ reasoning about smoking. Consequently, patients’ talk concerned only to a small extent their desire, ability, and need for smoking cessation and for taking steps toward making a commitment to stop smoking.

RNs’ communication strategies are central when focusing on the importance of smoking cessation. The patients should be involved in decision-making and in planning of their own individual interventions. To provide effective communication in smoking cessation with patients, improved education, and continuous training for RNs are needed.

Treatment plans with clear goals may help to structure objective communication and effective follow-up and also involve patients in shared decision-making, increasing their self-efficacy, and consequently their capability to quit smoking. Questions e-mailed before the consultation could be a first encouragement for patients to share decisions, take their own responsibility, reflect on their motivation and their ambivalence to quit smoking.

## Appendix

**Appendix A1:** Examples of the coding of registered nurses’ utterances and patients’ responses based on MITI scale and CLAMI categories

Nurse utterances (MITI)	Patient utterances (responses) (CLAMI)
So how do you anticipate the future, then? (Open question)	I will quit smoking. (commitment +)
How do you plan to go about to do that, it's a bit interesting to hear, will it happen or will it (Open question)	I think that it will evolve little by little, it will happen gradually. (other +)
That you decrease slowly. (Simple reflection)	Yes. (other+)
You have done really well as you have cut back by half, it is really good and that you have set your goals like ‘I do not smoke at work, I can go downtown without bringing the cigarettes. You have changed some of your habits’. (MI Adherent)	Yeah, that is a part of the general idea. (neutral)
It does not get so hysterical. (Complex reflection)	The cigarettes did influence a lot before. (neutral)
That is very good – excellent. (MI Adherent)	So, when some time will have passed … I will find that they are not that important after all. I will feel that these chewing gums or whatever nicotine replacement product I will chose, can replace the cigarette. (other+)
Do you smoke in the morning before you leave? (Closed question)	Yes, I do. (neutral)
It is not solely in the evening? (Closed question)	No, it gets to one cigarette in the morning. (neutral)
No, but you have to do it at your own speed and feel satisfied. Many feel worthless because ‘I have not been able to quit’, but it is pointless to think that way. It is better to think positively ‘look how good I am who has cut back this much’. (MI non-adherent)	But I do this just because you are telling me this now – otherwise I believe I would just quit. (neutral)
No, no, that doesn't make you feel any better, but imagine that you have cut back to 5 in half a year and then you are there and, I mean, for many people it takes time. It is better that it takes time and that you do it so that you can handle it. (MI non-adherent)	Yes, that one can chose one's own process. (neutral)
You know what it is like once you quit. Have I told you about that? (Closed question)	Mmm. (not coded)
I see a lot … I have had several who have relapsed. (See below, connected with the next nurse statement)	OK? (not coded)
When you have quit smoking you have more nicotine receptors in the brain and they switch off once you quit smoking. They do no harm, but they are of no use either. But if you smoke after half a year ‘I can do some smokes’ and then you will get a real abstinence problem and then you are stuck. I have had several patients who have kept up for eight months and then … it is very unnecessary. One should know that it is not possible; there are very few people who can smoke, smoke at parties, smoke just occasionally like that. They are not many, actually, so that one is aware of that. (MI non-adherent)	Mmm. (not coded) Yes, because there are those times that I have quit. (neutral)
Yes, you know. (not coded – the nurse is disrupted)	… and relapsed. That happens those times that one has taken … has been smoking at parties. (neutral)
When you are at a party, ‘well what does it matter’. (Complex reflection)	Perhaps, one loses judgement when one has had too much to drink. (neutral)
Everything is ok … one gets confident and so on. Now you will get to blow this again (spirometry). (not coded)	

MITI=Motivational Interviewing Treatment Integrity; CLAMI=Client Language Assessment in Motivational Interviewing.

**Appendix A2:** The MITI scale, global scores to characterize the entire interaction, rated in sex individual parameters

MITI global scores	
**Empathy** measures the extent to which the nurse attempts to ‘try on’ what the patients feel or think. Reflective listening is an important part of this characteristic. It is intended to capture all efforts that the nurse makes to understand the patient's perspective and convey that understanding to the patient. Empathy should not be confused with warmth, acceptance, genuineness, or patient's advocacy.		
*High, five on this scale:* The nurse approaches the consultation as an opportunity to learn about the patient. The nurse is curious and spends time exploring the patient's opinions and ideas about the target behavior.		
*Low, one on this scale:* The nurse shows indifference or active dismissal of the patient's perspective and experiences. There is little effort to gain a deeper understanding of complex events and emotions, and questions asked reflect shallowness or impatience.		
**Evocation** measures the extent to which the nurse understands the patient's motivation for change.		**MI-spirit** describes as collaborative, evocative, and honoring of patients autonomy and measures the general impressions of the three parameters Evocation, Collaboration and Autonomy/Support.
*High, five on this scale:* The nurse is curious about the patient's personal and unique ideas about why change is a good idea or not.		
The nurse understands the value of hearing the patient's own language in favor of change and actively creates opportunities for that language to occur.		
*Low, one on this scale:* The nurse has only a superficial interest in the patient's ambivalence or reasons for change and misses opportunities to explore these in detail.		
They nurse likely provides the patients with reasons for change, rather than eliciting them.		
**Collaboration** measures the extent to which the nurse behaves as if the communication is occurring between two equal partners.		
*High, five on this scale:* The nurse works cooperatively with the patient toward the goal. The nurse does not rely on dominance, expertise, or authority to achieve progress.		
The nurse is curious about the patient's ideas and is willing to be influenced by them.		
*Low, one on this scale:* The nurse does not work toward mutual understanding. The nurse relies on one-way communication based on own authority.		
and expertise for progress (in this study in smoking cessation), prescribing both the need for change and the means to achieve it.		
**Autonomy/support** is intended to convey the extent to which the nurse supports and actively fosters patient perception of choice.		
*High, five on this scale:* The nurse ensures that the topic of choice is raised during the session. The nurse views the patients as having the potential to move in the direction of health and they express optimism about the patients' ability to change.		
*Low, one on this scale:* The nurse views the patient as incapable of moving in the direction of health without input from the nurse.		
The nurse may insist that there is only one-way to approach the target behavior or may be pessimistic about the patient's ability to change.		
**Direction** scale measures the degree to which the nurse maintains appropriate focus on specific target behaviors. High scores on this scale do not necessarily reflect better use of MI.		
*High, five on this scale:* The nurse exerts substantial influence concerning the topic and course of the consultation. The nurse exerts direction by selectively reinforcing patient discussion toward the change with regard to the target behavior.		
*Low, one on this scale:* The nurse exerts little influence concerning the topic and course of the consultation. The consultation lacks structure and is aimless. Patients may discuss any topic of interest to them, without attempts by the nurse to focus on the troublesome behavior.		

MITI=Motivational Interviewing Treatment Integrity.

**Appendix A3:** The MITI scale, behavior code

MITI codes
***Questions*** contain the sub-codes **open and closed questions**. ***Closed questions*** are questions for which there is a particular answer, or for which only ***‘yes’*** **or** ***‘no’*** can be the reply. ***How long have you been smoking? Did you smoke this week?*** ***Open question**, * is coded when the nurse asks a question that allows a wide range of possible answers. The question seeks information, invites the patient's perspective, or encourages self-exploration. The open question allows the option of surprise for the questioner. ***Tell me more. Tell me about your nicotine cravings the past week**. *
***Reflections*** contain the sub-codes ***simple and complex reflections*** and capture reflective listening statements made by the nurse in response to patient statements. A reflection may introduce new meaning or material, but it essentially captures and returns to patients something about what they have just said. ***Simple reflections*** typically convey understanding or facilitate patient/nurse exchanges. These reflections add little or no meaning (or emphasis) to what patients have said. Simple reflections may mark very important or intense patient emotions but do not go far beyond the patient's original intent in the statement. ***Complex reflections*** add substantial meaning or emphasis to what the patient has said and give a deeper or more complex picture of what the patient has said. They may emphasize a particular part of what the patient has said to make a point or take the conversation in a different direction, or they may add obvious content to the patient's words, or they may combine statements from the patient to form summaries that are complex in nature. ***What have you already been told about how to handle your abstinence? (open question)*** ***Are you kidding? I have had nurse visits, I have all kinds of advice how to handle it, but I just don't do it, when the craving gets too strong. I smoke. Maybe I have a death wish*** ***You are pretty discouraged about this. (Reflection simple)*** ***You haven't been giving it your best effort yet. (Reflection complex)***
***Giving information*** is used when the nurse gives information, educates, provides feedback or discloses personal information. When the nurse gives an opinion, without advising, this category would be used. Giving information should not be confused with giving advice, warning, confrontation, or directing (MI-non-adherent behaviors) ***I talked to your wife and she said that she was really worried about your smoking**. *
***MI-adherent behaviors*** are behavioral consistent with a MI approach comprising asking permission before giving advice or information affirming the patient, emphasizing the patient's control, and supporting the patient. ***I have some information about how to reduce your abstinence and I wonder if I might discuss it with you**. *
***MI non-adherent behaviors*** could be advising without permission from the patient; confronting by arguing, correcting, blaming, criticizing, moralizing; directing by giving orders, commands, or imperatives. ***You said you shouldn't smoke but you smoke anyway***

MITI=Motivational Interviewing Treatment Integrity; MI=motivational interviewing.

**Appendix A4:** The CLAMI categories

CLAMI category
***Reason (R)*** with sub-codes Desire, Ability, and Need; usually refers to a specific rationale, basis, incentive, justification, or motive for making, or not making, the Target Behavior Change (TBC), and incorporates: -Patient discussions of health, family, or problems that are presented as a reason for considering change or not changing. -Patient expressions of worry and concern about their behavior and circumstances. -Statements incorporating the words ought, should, have to, or got to. **Benefits of a result of changing (+), as well as disadvantages of changing (−)**. ***My lungs are no good, so I have no choice (R***+***)**. * ***I just don't smoke that much (R***−***)**. * ***Desire** (d)* statements must have one of the following words: ‘want’, ‘desire’, ‘like’ or a close synonym or an antonym of them. ***I hate being an addict (R***+***d), I want to stop smoking (R***+***d), I'd like to quit (R***+***d)**. * ***Ability (a)*** statements include the word ‘can’, ‘possible’, ‘willpower’ or ‘ability’ or a close synonym or antonym of them. ***I just can't quit (R***−***a), I can quit (R***+***a)**. * ***Need (n)*** statements have to include some form of the words ‘need’ or ‘must’. ***I need to stop smoking (R***+***n), I must quit (R***+***n)**. *
***Other (O)*** is intended to allow coders to capture language that clearly reflects the patient's movement toward change, but does not necessarily fit easily into the Reason category as general statements of problem recognition. Similarly, minimization of problems and hypothetical language will also be categorized here. ***If my wife would stop pushing me, I know I would quit (O***+***). If I threw away all of my cigarettes I'd be less tempted to smoke (O***+***). My daughter has told me: If you quit smoking Mum you won't ever need to buy me a birthday present (O***+***)**. *
***Taking steps (T/S)*** includes concrete and specific steps the patient has taken toward the behavior change. These statements usually describe a particular action that the patient has done in the very recent past that is clearly linked to moving toward or away from TBC. Taking Steps represents the only time that past patient language is given a code. ***I didn't smoke at all last week (T/S). I have told all my friends that I will stop smoking next Friday (T/S)**. *
***Commitment (C)*** reflects motivating factors related to change as an agreement, intention, or obligation regarding future TBC. Commitment can be expressed directly via a committing verb, or indirectly. Patient statements of how they will rearrange their life in the future relating to the TBC are considered commitment statements. ***No way I'm going to stop smoking (C***−***). I'm going to do it (C***+***). I threw away all of my cigarettes (C***+***)**. *
***Follow/Neutral (F/N)*** gives no indication of patient inclination either toward or away from the TBC. The patient may be asking a question, making noncommittal statements, saying TBC-irrelevant things, or just following along with the conversation. Note that a patient turn is coded at Follow/Neutral *only* if it contains no other code able utterance and that only TBC-relevant change talk is coded. ***Sure (F/N). Ok (F/N)**. *

CLAMI=Client Language Assessment in Motivational Interviewing.
